# Clinical and Genetic Factors to Inform Reducing Colorectal Cancer Disparitites in African Americans

**DOI:** 10.3389/fonc.2018.00531

**Published:** 2018-11-20

**Authors:** John M. Carethers

**Affiliations:** Division of Gastroenterology, Departments of Internal Medicine and Human Genetics, Rogel Cancer Center, University of Michigan, Ann Arbor, MI, United States

**Keywords:** colorectal cancer, African American, cancer disparity, colon cancer prevention, colon cancer risk, colon cancer genetics, colon cancer immunology, colon cancer survival

## Abstract

Colorectal cancer (CRC) is the third most prevalent and second deadliest cancer in the U.S. with 140,250 cases and 50,630 deaths for 2018. Prevention of CRC through screening is effective. Among categorized races in the U.S., African Americans (AAs) show the highest incidence and death rates per 100,000 when compared to Non-Hispanic Whites (NHWs), American Indian/Alaskan Natives, Hispanics, and Asian/Pacific Islanders, with an overall AA:NHW ratio of 1.13 for incidence and 1.32 for mortality (2010-2014, seer.cancer.gov). The disparity for CRC incidence and worsened mortality among AAs is likely multifactorial and includes environmental (e.g., diet and intestinal microbiome composition, prevalence of obesity, use of aspirin, alcohol, and tobacco use), societal (e.g., socioeconomic status, insurance and access to care, and screening uptake and behaviors), and genetic (e.g., somatic driver mutations, race-specific variants in genes, and inflammation and immunological factors). Some of these parameters have been investigated, and interventions that address specific parameters have proven to be effective in lowering the disparity. For instance, there is strong evidence raising screening utilization rates among AAs to that of NHWs reduces CRC incidence to that of NHWs. Reducing the age to commence CRC screening in AA patients may further address incidence disparity, due to the earlier age onset of CRC. Identified genetic and epigenetic changes such as reduced *MLH1* hypermethylation frequency, presence of inflammation-associated microsatellite alterations, and unique driver gene mutations (*FLCN* and *EPHA6*) among AA CRCs will afford more precise approaches toward CRC care, including the use of 5-fluorouracil and anti-PD-1.

## Introduction

Colorectal cancer (CRC) is common in the U.S. It is the third most prevalent cancer (behind lung and prostate in men and behind lung and breast in women), but the second most deadliest cancer in both men and women (1). Among categorized races and ethnic groups in the U.S., African Americans (AAs) demonstrate the highest incidence among males and females for CRC (56.4 and 41.7 per 100,000 population, respectively) and the highest mortality among males and females (25.1 and 16.5 per 100,000 population) over Caucasians, Asians, Hispanics, and American Indian/Alaskan Natives. The incidence and mortality among AAs compare unfavorably to the U.S. general population, where rates for incidence are 45.9 and 34.8 per 100,000 and rates for mortality are 17.3 and 12.2 per 100,000 population (1). The ratio for CRC incidence between AAs and Non-Hispanic Whites (NHW) is 1.13, meaning that for every 100 CRCs in NHWs, there are 113 CRCs in AAs (2). This disparity in incidence is further amplified when one examines CRC mortality, with the AA:NHW mortality ratio of 1.32, meaning that for every 100 CRC deaths in NHWs, there are 132 deaths in AAs (2). Recent data regarding the observed increase of CRC among young patients (< 50 years of age) suggests that the disparity begins early for AAs with rates for CRC incidence at 7.9 per 100,000, compared to NHWs and Asians of 6.7 and 6.3 per 100,000, respectively, among patients aged 20–44 (3, 4). It should be noted that the overall trends for CRC death have decreased for both AAs and NHWs since the 1990s; however, for males, the decrement has been faster for NHWs than AAs increasing the disparity, whereas for females the decrement in CRC mortality has be roughly equivalent for NHWs and AAs, maintaining a constant but stable mortality disparity (5). AAs tend to present with less localized and regional staged CRC and more distant staged CRC when compared to Caucasians (localized 38 vs. 39%, regional 32 vs. 36%, distant 25 vs. 21%) (1). Furthermore, AAs with CRC show an overall survival rate for all stages of 58% at 5 years compared to 66% for Caucasians. When further broken down, AAs show reduced survival than Caucasians at all stages (localized 86 vs. 90%, regional 65 vs. 72%, distant 10 vs. 14%) (1).

**Figure 1:**
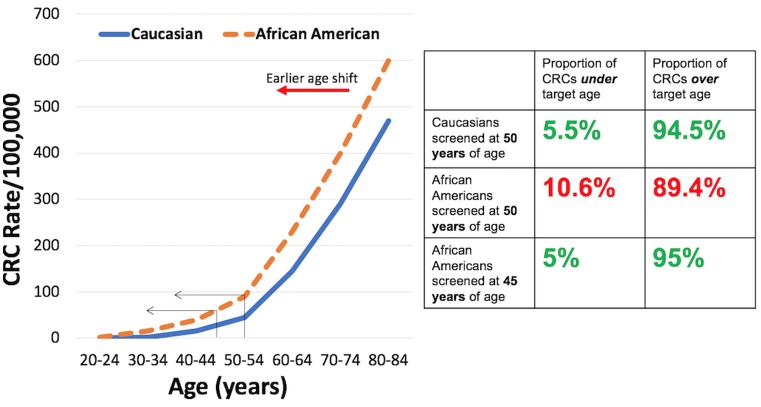
**(Left)** Rates per 100,000 of colorectal cancer for Caucasians and African Americans by age group. **(Right)** Proportion of colorectal cancers under and over targeted ages for screening for Caucasians and African Americans.

Why is there a disparity in CRC incidence and mortality for AAs? The answer is likely multifactorial. There are a number of modifiable and non-modifiable factors that can change the risk of developing and dying from CRC, with some adversely affecting risk and some reducing risk. Modifiable risks include: diet and intestinal microbiome composition, socioeconomic factors, screening utilization rates, healthcare access, education level, physical activity, use of tobacco products, use of alcohol, and use of aspirin/NSAIDs and hormonal replacement therapy, among others (6, 7). The modifiable risks have been shown to influence CRC incidence at the epidemiological and individual levels, with some measures that change modifiable risks hard to implement consistently in individuals among various populations. Non-modifiable risks include: age, family history, and racial background (6). These non-modifiable risks infer genetic causes as part of the driver for CRC risk. In particular, age is a strong predictor for the development of adenomas, a precursor of CRC, and CRC itself (6). There is a near exponential rise in CRC in the U.S. population around age 50 years (and thus this age was selected to commence CRC screening in average risk individuals), with 94.5% of all CRCs occuring after this age and 5.5% occuring younger than age 50 years (8). For AAs, the CRC rate curve is shifted to earlier ages (6, 9–12) such that the proportion of CRCs are doubled under the age of 50 years compared to Caucasians (10.6 vs. 5.5%) (Figure 1). With screening commencing at age 45 years for AAs (13), the proportion of CRCs under age 45 is the same proportion of CRCs for Caucasians starting screening at age 50 years (Figure 1) (6).

For most CRCs, adenomas are the direct precursor (14, 15). Thus, with an increased rate of CRC among AAs, there should be some evidence of increased adenomas, and indeed there is. Lieberman *et al*. reported the prevalence of high risk adenomas (those >9 mm in diameter), in AAs (7.7%) and Caucasians (6.2%) among 85,000 individual colonoscopies (16). In a follow-up study of 327,785 average risk adults, both male and female AA patients demonstrated higher ratios of high risk adenomas for nearly all 5-year age subgroups (e.g., 50–54, 55–59 years, etc.) that increased with age (17). To match the observed 7–15% higher proximal distribution of CRCs among AAs compared to Caucasians (6), proximal high risk adenomas are more prevalent among AAs than Caucasians, with odds ratios of 1.26 (18) and 1.15 (17) in two separate studies that became significant after the age of 60 years. This higher prevalence of adenomas proximal to the colonic splenic flexure in AAs likely contributes to the disparity because colonoscopy, the gold standard tool for screening in the U.S., is not as effective in reducing mortality from right-sided lesions (19–21). The combination of a proximal, harder-to-detect lesions coupled with reduced ability to detect right-sided lesions with any screening test amplify the magnitude of the disparity (6). At present, there is no evidence that there is increased prevalence of high risk sessile serrated adenomas in AAs compared with Caucasians (0.3 vs. 0.2% of colonic lesions, *p* = 0.71) as a component of the increased right-sided lesions (22).

Below, we explore some specific additional modifiable and non-modifiable risks that may inform approaches to reduce the CRC disparity among AAs. Some of these risk parameters have been investigated, and some interventions that address specific parameters have proven to be effective in lowering the disparity. Others may lead to more precise approaches toward CRC prevention and care.

## Environmental factors

There is growing evidence that diet and gut microbiome composition greatly influence adenoma and CRC risk (6, 23, 24); however, the data is scant with regards to specific racial differences. Sulfidogenic bacteria produce hydrogen sulfide, which triggers pro-inflammatory pathways and hyperproliferation, was shown more abundant among AA CRC patients from uninvolved colon biopsies compared to NHW CRC patients (25). Proinflammatory *Fusobacterium nucleatum* and *Enterobacter* species were found significantly higher among AAs as compared to NHWs at screening colonoscopy, with AAs demonstrating decreased microbial diversity (26). O'Keefe et al. explored 2-week food swaps with before and after colonic mucosa biopsies in AAs and rural Africans, whose diet typically contained a high-fat and low-fiber Western-style diet (AAs) vs. a high-fiber, low-fat African-style diet (rural Africans) (27). Compared to mucosal biopsies before the diet exchange, post-diet swap biopsies showed reciprocal changes in biomarkers. In particular, AAs after 2 weeks of the African-style diet lowered the proliferation marker Ki67 by 50%, whereas rural Africans after the Western-style diet nearly doubled colonocyte proliferation. Furthermore, AAs showed reduced intraepithelial lymphocytes after the African-style diet, whereas rural Africans on the Western-style diet increased inflammation. The short chain fatty acid butyrate, a normal fuel molecule for healthy colonocytes that is made by gut microbes, increased in the colons of AAs on the African-style diet whereas the secondary bile acid deoxycholic acid, which may be a carcinogenic compound, decreased. The opposite happened in the rural Africans' colons after 2 weeks of the Western-style diet, with decreased butyrate levels but increased deoxycholate levels (27). These observations strongly suggest that even short-term diet manipulation can modify colonic contents and colonocyte proliferation parameters that may influence risk for CRC. Extrapolation for prolonged Western diet exposure matches epidemiological evidence for this type of diet and strong association with CRC development. The implications by the O'Keefe et al. data indicate that even higher risk populations for CRC such as AAs might be able to reduce that risk with diet manipulation. This would likely require a lifestyle change that is sustained over long periods of time (years) to observe the risk reduction.

## Genetics

Inherited germline adenomatous polyposis syndromes such as familial adenomatous polyposis, *MYH*-associated polyposis, polymerase proofreading associated polyposis, Lynch syndrome, and Familial Colorectal Cancer Type X seem to exist in multiple racial and ethnic populations including AAs, but there is no evidence for a predilection for AAs for any of these syndromes (9, 28). Guindalini et al. examined AA Lynch syndrome families, and showed that two-thirds of the families contained a germline mutation in the DNA mismatch repair gene *MLH1*, with a cumulative cancer risk similar to those from European descent (29). However, multiple novel mutations within *MLH1* were discovered in AAs that were not demonstrated in national mutational databases, suggesting genetic diversity of the mutational spectrum of *MLH1*.

Somatic mutations of key cell regulatory genes to inactivate their growth regulatory abilities are a defining property of sporadic CRCs. CRCs can be segregated into hypermutated tumors (with hundreds to thousands of accumulated mutations) that are the result of failed DNA mismatch repair (typically by hypermethylation of *MLH1*) or mutation in the *POLE* gene that encodes polymerase ε, and non-hypermutated tumors (with 1–8 accumulated driver mutations) (30). While each individual's CRC has an overall unique mutational profile (15), common to hypermutated CRCs are a spectrum of accumulated somatic mutations that are largely the result of frameshift mutations in genes with coding microsatellites, such as *ACVR2, TGFBR2, MSH3*, and *MSH6*, along with *BRAF* mutations (30). Non-hypermutated CRCs commonly demonstrate mutations in *APC, TP53, KRAS, TTN*, and *PIK3CA* (30). This large and defining dataset could not determine any racial differences in gene mutations due to the paucity of AA CRCs examined (< 5 of 224 primary CRCs) (30).

Guda et al. (31) sequenced 103 AA and 129 Caucasian CRCs to identify any AA-specific somatic driver mutations. Three genes were found exclusively mutated in CRCs from AAs: *EPHA6* (mutational frequency of 5.83% in AAs, 0% in Caucasians), *FLCN* (mutational frequency of 2.91% in AAs, 0% in Caucasians), and *HTR1F* (mutational frequency of 2.91% in AAs, 0% in Caucasians) (31). Further examination of mutations in *EPHA6* showed missense and splice site mutations, and mutations in *FLCN* were frameshift insertions and non-sense mutations, identifiying most of the discovered mutations as deleterious (31). These exclusive mutations among AA tumors raise the possibility that *EPHA6* and *FLCN* are unique driver genes in this population, but it remains to be determined if these mutated genes in any way contribute toward the incidence or mortality disparity observed.

Inactivation of the DNA mismatch repair gene *MLH1* is the (epi)genetic cause for most hypermutated CRCs. Hypermutated tumors are more often located in the proximal colon, demonstrate microsatellite instability (or MSI-High, a biomarker for ongoing frameshift mutation), and demonstrate lymphoid aggregates and increased intraepithelial lymphocytes within the CRC as a reaction to immunologically-driven frameshifted neoantigen proteins (15, 32). Patients with hypermutated tumors demonstrate longer survival as compared to patients with non-hypermutated tumors (15, 33) without any treatment, and further demonstrate improved survival with immune checkpoint inhibitor therapy (34, 35). Patients with hypermutated tumors also respond less to standardized 5-fluorouracil-containing therapy (5-FU), as DNA mismatch repair is one mechanism to execute the toxicity of 5-FU (36–43). In a population-based study, the proportion of Caucasian CRCs that demonstrated MSI-High (hypermutated) was 14 vs. 7% among AA CRCs (44). A meta-analysis of the few MSI-High studies that identified race in cohorts calculated an odds ratio of 0.78 for AA CRCs to demonstrate MSI-H as compared to Caucasian CRCs, but with few studies available did not reach a statistical significance (45). The apparent lower hypermutated CRC prevalence among AAs may contribute to decreased survival as a group, and may further limit the use of immune checkpoint inhibitors that could prolong survival. Lowered MSI-High prevalence among AAs might also mean a higher proportion may respond to 5-FU treatment.

Another mechanism to inactivate DNA mismatch repair somatically is through inflammatory pathways. Tseng-Rogenski et al. demonstrated that oxidative stress and IL-6, both released when inflammation is present, shifts the DNA MMR gene MSH3 from the nucleus where it repairs DNA to the cytosol, where it cannot repair DNA (46, 47). MSH3 normally hetrodimerizes with MSH2 to form MutSβ that repairs slippage at DNA microsattelite repeats that contain two (di), three (tri), or four (tetra) or more nucleotides (33, 48). When MutSβ becomes non-functional with the subcellular shift of MSH3, microsatellite slippage mutations accumulate and can be detected as “elevated microsatellite alterations at selected tetranucleotide repeats” or EMAST. CRCs manifesting EMAST tend to have intratumoral and intraepithelial inflammation (showing intimate association between immune cells and EMAST), be staged as advanced cancers with higher frequency of metastases, and patients show poor survival (49–52). Devaraj et al. showed that 49% of AA rectal cancers demonstrated EMAST as compared to 26% of Caucasian rectal cancers (53), linking this aggressive-associated biomarker as a potential contributor to the disparity. Lowering inflammation may be a possible avenue to reduce these inflammatory-associated microsatellite alterations and possibly improve outcome (54).

## Immune surveillance

Immune cells play key roles in cancer pathogenesis and patient outcome (55). Galon et al. showed that high levels of lymphocytes at the center and invasive margin of the tumor irrespective of stage was associated with improved patient survival compared to those patients with low levels of lymphocytes (56). These lymphocytes were mainly CD8^+^ and granzyme B^+^ cytotoxic T lymphocytes, as well as CD45RO^+^ memory cells (57). MSI-High CRCs induce inflammation by generation of expressed peptides from frameshifted genes that are recognized as neoantigens by the immune system (15, 32) and induce memory T cell differentiation with tumor cells acquiring PD-1 receptors (57); however, it is the CD8^+^/CD45RO^+^ T cell immune response irrespective of being an MSI-High or microsatellite stable (MSS) tumor that may be a more over-riding determinant of patient outcome (57). Patients with MSI-High tumors no matter their organ origin are now eligible for anti-PD-1 therapy as approved by the U.S. Food and Drug Administration (35).

The lower frequency of MSI-High among AA CRC patients means that as a group, there will likely be less benefit from anti-PD-1 therapy (44). Although the number of CD8+ T cells are demonstrated higher among MSI-High CRCs, AA MSI-High and AA MSS CRCs lack high counts of CD8^+^ T cells as compared to Caucasian MSI-High and MSS CRCs (44). In a study involving 250 CRCs, Basa et al. showed reduced intraepithelial and intra-tumoral granzyme B^+^ T cells among AA MSS CRCs as compared to Caucasian MSS CRCs (58). These observations of lower CD8^+^ and granzyme B^+^ T cells among AA CRCs implicate less immune cytotoxicity for tumor cells as a potential contributor to poor outcome and the observed disparity.

## Prevention

CRC Screening is effective in reducing morbidity and mortality from CRC, and is highly cost effective (6). There have been several proposed strategies to reduce the disparity for CRC in AAs with all of them relying on improved prevention (59). Patient education might address patient-level barriers for CRC screening, but there are challenges in the ability to effectively reach all target populations. Physician education might address the lower rates of physician recommendation for screening in AAs, but there is no data on effectiveness of this strategy as well as issues of the target physician population (e.g., gastroenterologists, primary care physicians, etc.). Patient navigation provides strong evidence for increasing CRC screening rates for AAs and is cost-effective. However, this requires training and implementation, and there are barriers to cost and insurance coverage. Another strategy is to lower the age for screening (see Figure 1) as recommended by the U.S. Multi-Society Task Force on Colorectal Cancer to 45 years of age (13). This approach should reduce the burden of early disease, but there has been no prospective study on its effectiveness (59). However, this is the first time race is considered in these nationally-influential multi-society guidelines.

There is strong evidence that increasing CRC screening reduces the disparity for AAs (as well as reduce the overall incidence of CRC). The Delaware Cancer Consortium performed 10,000 patient navigations for colonoscopic CRC screening among AAs and Caucasians between 2001 and 2009 (60). The stage at diagnosis for AA CRC patients change dramatically between 2001 and 2009 with the intervention of navigated CRC screening, with distant disease dropping from 23 to 7%, regional disease dropping from 56 to 33%, and local disease (potentially curable) rising from 15 to 50% (60). Additionally, as the number of navigated patients screened increased (AA: 47.8 to 73.5%; Caucasians: 58.0 to 74.7%), the CRC incidence dropped as the study progressed for both AAs and Caucasians, with the AA rate falling from 68 to 48 per 100,000, and Caucasians falling from 60 to 48 per 100,000 (60). This means the disparity in CRC incidence that existed at the beginning of the study was no longer by the end of the study. Similarly, CRC mortality was reduced over the course of the study for AA CRC patients, dropping from 31.27 to 18.35 per 100,000, and Caucasians dropped from 19.45 to 16.94 per 100,000 (60). The AA CRC mortality closed a large gap disparity such that by the end of the study, the rate approximated that observed for Caucasians. These data strongly suggest that increasing the rate of CRC screening overall for a population can reduce disparity.

## Summary and the future

There is disparity in CRC incidence and mortality for AAs that likely has multifactorial causes. AAs present with earlier onset, higher proportion of proximal, and higher proportion of young CRC. Similarily, AAs show higher proportions of advanced and proximal adenomas, the precursors to CRC. Westernized diets lowers short chain fatty acids and increases secondary bile acids, and is a likely a contributor. Somatic genetic biomarkers such as lower frequency of MSI-High and higher frequency of EMAST, and potentially unique driver genes such as *FLCN* and *EPHA6* may play a role for the disparity among AA CRC patients. Impaired immune response via lower CD8^+^ and/or granzyme B^+^ T cells to control tumor growth or spread may also contribute (see Table 1). Prevention through CRC screening is a key component to reduce the disparity.

**Table 1:** Unique genetic and biomarker findings in African American colorectal cancers.

**Genetic finding or biomarker in African American CRCs**	**Resulting outcome compared with Caucasian CRCs**	**References**
Decreased frequency of MSI-High	Poor survival, less likely to respond to PD-1 checkpoint inhibitors	(44, 45)
Increased frequency of inflammatory-associated microsatellite alterations or EMAST	Increased metastasis, poor survival	(53)
Somatic *FLCN* mutation	New potential driver gene	(31)
Somatic *EPHA6* mutation	New potential driver gene	(31)
Somatic *HTR1F* mutation	New potential driver gene	(31)
Decreased high numbers of CD8^+^ T lymphocytes	Increased metastasis, poor survival	(44, 58)
Decreased numbers of granzyme B^+^ T lymphocytes	Increased metastasis, poor survival	(58)

There is great possibility of other modifiable and non-modifiable causes of CRC can be manipulated to reduce CRC disparity for incidence or mortality among AAs. An understanding will be enhanced through collection of racially diverse biorepository materials and increased participation in clincial trials for CRC prevention and treatment. Some interesting questions that arise include: (1) what is the effectiveness of screening AAs beginning at the age of 45 years? (2) why do advanced adenomas present earlier in AAs? (3) why do AAs possess more right-sided adenomas (and CRCs)? (4) are there other somatic genetic differences? (5) what if anything might suppress immune function within AA CRCs? Strategies to answer these and other questions can further address the disparity among AA CRCs (61).

## Author contributions

The author confirms being the sole contributor of this work and has approved it for publication.

### Conflict of interest statement

The author declares that the research was conducted in the absence of any commercial or financial relationships that could be construed as a potential conflict of interest.

## Abbreviations

MSI-High: microsatellite instability-high
MSS: microsatellite stable
EMAST: elevated microsatellelite alterations at selected tetranucleotide repeats
MMR: DNA mismatch repair
CRC: colorectal cancer
AA: African American
SEER, surveillance, epidemiology: and end results program
NSAID: non-steroidal anti-inflammatory drug
HRT: hormone replacement therapy.
